# Wnt Signaling in Cancer Stem Cell Biology

**DOI:** 10.3390/cancers8070060

**Published:** 2016-06-27

**Authors:** Felipe de Sousa e Melo, Louis Vermeulen

**Affiliations:** Laboratory of Experimental Oncology and Radiobiology (LEXOR), Center for Experimental Molecular Medicine (CEMM), Academic Medical Center (AMC), Amsterdam 1105AZ, The Netherlands; felipedesousamello@gmail.com

**Keywords:** Wnt signaling, cancer, stem cells, cancer stem cells, cancer stem cell niche, Wnt inhibition, targeted therapy

## Abstract

Aberrant regulation of Wnt signaling is a common theme seen across many tumor types. Decades of research have unraveled the epigenetic and genetic alterations that result in elevated Wnt pathway activity. More recently, it has become apparent that Wnt signaling levels identify stem-like tumor cells that are responsible for fueling tumor growth. As therapeutic targeting of these tumor stem cells is an intense area of investigation, a concise understanding on how Wnt activity relates to cancer stem cell traits is needed. This review attempts at summarizing the intricacies between Wnt signaling and cancer stem cell biology with a special emphasis on colorectal cancer.

## 1. Introduction

Dedicated populations of stem cells ensure self-renewal of most adult epithelial tissues [1]. These cells reside in specialized niches where they integrate various environmental and intrinsic signaling inputs to coordinate cell fate determination and maintain tissue homeostasis. The ability of these stem cells to indefinitely self-renew makes them prime candidates for accumulating mutations that may lead to tumor initiation [2]. Cancers have long been acknowledged to present as heterogeneous entities. Not all cells within a particular malignancy are deemed equal with respect to their biological qualities. Most importantly, the tumor initiation capacity seems to be restricted to a small population of tumor cells that continuously fuel tumor growth [3]. Because these cells are endowed with self-renewal capacity and multi-differentiation potential, they are often referred to as cancer stem cells (CSCs). Interestingly, the biology of normal and CSCs within the same tissue is highly interrelated. This is evident from the fact that molecular signals that define and maintain normal stem cells are often aberrantly activated in tumor cells originating in the same organ. A prime example, and the main topic of our review, is the role of Wnt signaling in defining CSC features across several malignancies with a particular emphasis on colorectal cancer (CRC). We summarize the latest advances on the intrinsic and extrinsic regulation of Wnt signaling and describe a model in which incremental levels of pathway activities shape a hierarchical organization of both normal and cancerous tissues. This poses an interesting conundrum on the rational design of drugs aimed at depleting the CSCs within tumors while sparing the function of normal tissues. We will discuss the most recent therapeutic developments aimed at targeting components of the Wnt signaling pathway and how this may impact the maintenance of the CSC population and ultimately the clinical outcome.

## 2. Wnt Signaling in Cancer

### 2.1. Colorectal Cancer

**Figure 1:**
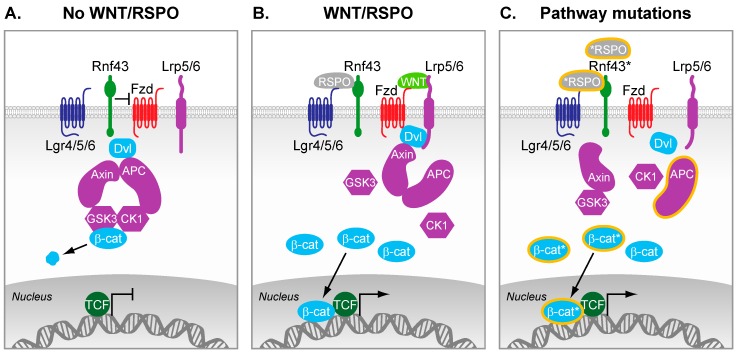
The Wnt signaling pathway. (**A**) In the absence of Wnt ligands and R-spondins, β-catenin is kept under low cytosolic levels by the destruction complex. This complex contains Axin2, Adenomatousis Polyposis Coli (APC), and the kinases Casein kinase 1 (CK1) and GSK3-β, which altogether primes β-catenin for proteasomal degradation; (**B**) R-spondins bind to Lgrs and RNF43, which results in the stabilization of Fzd receptors. Wnt ligands bound to Fzd and LRP5/6 co-receptors trigger formation of Dvl-Fzd complex and subsequent destabilization of the destruction complex. β-catenin is therefore stabilized, can accumulate in the cytosol, and subsequently translocates in the nucleus where it converts T cell-factor (TCF) into a transcriptional activator. In intestinal stem cells, this nuclear β-catenin bound to TCF enables the efficient transcription of genes that are important regulators of stem cell fate (e.g., Leucine-Rich Repeat Containing G Protein-Coupled Receptor 5 (*LGR5*), Achaete-scute complex homolog 2 (*ASCL2*)); (**C**) Truncating mutations in APC are frequently observed in colorectal cancer (CRC). As a result, the destruction complex cannot properly form, which results in inefficient targeting of β-catenin for degradation. Therefore, β-catenin can accumulate and form active transcription factor complexes with TCF proteins in the nucleus, even in the absence of external signals. Additional reported mutations of various pathway components lead to constitutive activation of the pathway (mutations and truncations are depicted by a * or orange lining).

Over the past decade, the mutational landscape of CRC has unfolded and it became apparent that most, if not all, CRCs display genetic alterations in the Wnt pathway. Although the nature of these mutations may be distinct, they ultimately result in the stabilization of β-catenin, the key transducer of canonical Wnt signals. The most prevalent mutations are truncating events that inactivate the tumor suppressor Adenomatousis Polyposis Coli (APC) [4], a protein that binds and stabilizes AXIN2 and Glycogen synthase kinase 3 β (GSK3β) to form the β-catenin destruction complex (Figure 1). The latter is responsible for maintaining low cytosolic levels of β-catenin by directing it for proteasomal degradation. Interestingly, additional Wnt activating mutations have been reported that display mutual exclusivity with inactivating APC truncation. First and foremost is the identification of β-catenin stabilizing mutations that result in its constitutive nuclear localization and target gene transcription [4]. Furthermore, recent RNA-sequencing efforts have identified multiple fusion transcripts including recurrent gene fusions involving R-spondin family members RSPO2 and RSPO3 that together occur in 8% of colon tumors [5]. R-spondins are secreted proteins that potentiate Wnt signaling in a Wnt ligand dependent manner and act as oncogenic drivers in RSPO fusion tumors [5,6]. Finally, a small subset of CRC displays inactivation of *RNF43* that encodes an E3 ubiquitin ligase that negatively regulates Wnt signaling [7,8].

As mentioned above, Wnt activating mutations occur early during colon tumorigenesis whereas the progression of the disease is often accompanied by other genetic alterations, most commonly seen in *KRAS*, *BRAF*, *TP53,* and *SMAD4* [5,9,10]. Although these alterations are recurrently described as driver mutations in various cancers, it is still unknown which of these are required to maintain established tumors and whether interfering with Wnt signaling might be a viable therapeutic target in the background of additional drivers.

In a recent elegant mouse study, Dow et al. [11] addressed this question by using doxycycline inducible short hairpin RNA (shRNA)-mediated silencing and reactivation of *APC*. Administration of doxycycline resulted in activation of Wnt signaling, intestinal hyperproliferation, and a block in differentiation. Importantly, this phenotype was completely reversible when endogenous levels of APC were restored after doxycycline removal. Of note, this was also true even in the presence of other CRC canonical oncogenic mutations, suggesting that APC loss and hyperactivation of Wnt signaling is crucial for tumor maintenance [11].

In a separate study, a functional blockade of R-spondins in RSPO fusion tumors caused tumor regression even in the presence of mutations in *KRAS*, *BRAF*, and/or *SMAD4* [6]. Altogether this suggests that Wnt signaling activity remains a major oncogenic driver in CRC despite the presence of genetic instability and supports the continuing efforts of the research community in bringing relevant Wnt pathway inhibitors to the clinic. Of note, it was reported that in different types of CRC precursor lesions distinct types of Wnt activating mutations occur and at a histopathologically different stage. For example, in conventional tubular adenomas inactivating *APC* mutations are very early events whilst in serrated neoplasia *RNF43* mutations are selected for at a much later stage [12]. Thus, different context select for distinct mutational spectra, also with respect to Wnt signaling, and are accompanied with distinct biological and clinical presentation.

### 2.2. Other Cancers

The role of Wnt signaling in carcinogenesis extends beyond CRC. Mutations of pathway components are also frequently detected in a subset of hepatocellular carcinoma. There, hyperactivation of Wnt signaling is mediated by loss of function and inactivation of the Wnt negative regulators *AXIN1* and/or *AXIN2* [13]. Early studies in mouse mammary tumor development identified int1 insertion as a frequent target for insertional activation by the mouse mammary tumor virus (MMTV) [14]. Soon after, the mouse int-1 gene was recognized to be part of a substantial group of mammalian genes, now commonly known as Wnt ligands. Hyperactivation of Wnt signaling in breast cancers is rarely due to mutations in the pathway but rather a consequence of increased ligand production. Autocrine production of various Wnt ligands was reported in a subset of breast and ovarian cancer cell lines, which was shown to lead to an increase in beta-catenin stability [15]. Importantly, this increase in Wnt signaling activity could be blocked by overexpression of the receptor-Wnt ligand antagonist Secreted frizzled-related protein (SFRP) or Dickkopf-related protein 1 (DKK1), resulting in a decrease in Wnt signaling activity and reduced cell proliferation. Indeed, epigenetic silencing of SFRP1 and Wnt inhibitory factor 1 (Wif1) has been reported in a significant amount of human breast cancers and was also shown to correlate with a poor disease outcome [16]. Another example of a Wnt driven tumor is medulloblastoma, one of the most common juvenile brain tumors. Medulloblastoma is a heterogeneous disease that can be subdivided into distinct molecular subgroups that present with different clinical outcomes. At least 10% of all medulloblastoma are classified as the so-called Wnt subtype since they exhibit a high frequency of β-catenin activating mutations [17]. Patients comprised within the Wnt subtype often display a relatively good clinical outcome. Interestingly, this is in contrast with the Myc-driven subgroup of medulloblastoma that generally display a more dismal prognosis [17]. Since Myc is commonly described as a Wnt canonical target, additional features beyond Wnt signaling activation are likely contributing to the heterogeneous clinical behavior of the aforementioned tumor subtypes.

It is intriguing to note that both the type of Wnt activating mutations and the level of activation of the pathway seem to be selected in a tissue specific manner. For example, germline *APC* truncation causes an inherited autosomal condition called Familial Adenomatousis Polyposis (FAP), which almost exclusively results in an increased risk in developing colon cancer but with only a limited increase in the incidence of other malignancies [4]. Conversely, WTX mutations that also result in a deficient β-catenin destruction complex are only occasionally observed in CRC but more frequently associated with pediatric renal cancer (nephroblastoma) [18]. As we will discuss below, fine-tuning of Wnt activity is essential to optimally transform neoplastic cells and the level of Wnt activation required for transformation is likely to be tissue and cell type specific [19].

## 3. Wnt Activity and Stem Cell Function in Cancers

### 3.1. Tumor Initiation

Cancer develops from a single founding cell, the identity of which is still highly disputed. Self-renewal capability of most adult tissues is restricted to stem cells and therefore these cells are likely to be a prime target for cellular transformation. In addition, adult stem cells are usually long-lived, which is a prerequisite to accumulate sufficient genetic events, especially in the case of epithelial tissues undergoing high turnover. Support for the stem origin of intestinal cancer comes from mouse models of intestinal tumorigenesis that have confirmed that adenomas develop preferentially when loss of *Apc* is induced in the stem cell compartment [20]. More recently, the functional consequence of mutations in intestinal stem cells was unveiled using quantitative models. Previous studies on normal intestinal homeostasis had shown that intestinal crypts become monoclonal over time, following a pattern of neutral drift dynamics in which functionally equivalent stem cells either expand or disappear stochastically until they either take over the crypt or are lost [21,22]. If one Intestinal stem cell (ISC) acquires a neutral mutation that does not confer any selective advantage, this targeted cell has a high risk of being replaced by a normal stem cell within the crypt. In contrast, the probability for an *Apc* mutation, for example, to become fixed—that is, to enable that mutated stem cell to populate the entire crypt—is ~60%, which still means that in a large fraction of cases the mutated cell will be replaced by one of its wild-type stem cell neighbors and will be lost [23]. For the first time, such studies have enabled quantitative measurements of the competitive advantage stem cells are endowed with when a mutational event occurs [23,24]. Unfortunately, these dynamics have only been defined when mutations are introduced in stem cells and to date no data have established the fitness mutations may confer in more differentiated cell types. This is of relevance since multiple lines of evidence in the gut have shown that tumors do not necessarily arise from stem cells and dedifferentiation of intestinal enterocytes can occur and lead to intestinal tumors. For example, enforced activity of both Wnt signaling and NFκB in differentiated enterocytes is required to initiate adenomas from these cells and is accompanied by the re-expression of genes present in the intestinal stem cell signature [25]. More recently, Gremlin1 overexpression in mice was shown to result in the formation of ectopic crypts in intestinal villus. These ectopic crypts could persist long enough to acquire additional mutations that would ultimately lead to neoplasia [26]. Another recent example has been described for the case of terminally differentiated small intestinal tuft cells, in which *Apc* deletion is not sufficient to induce tumorigenesis [27]. Only in the presence of inflammation, that could be initiated even several months after induction of the mutation, these *Apc*-deficient tuft cells could form colonic tumors [27]. Altogether, this exemplifies how mutations in non-stem cells may enable these cells to persist long enough to acquire secondary mutations that lead to tumor formation. Altogether it still remains to be established whether it is the long-lived nature of stem cells or particular intrinsic features of these cells that makes them more susceptible to transformation. We recently reported that stem cell associated expression of anti-apoptotic molecules contributes to the ability of intestinal stem cells to efficiently transform [28]. Expression of *Bcl-2*, which is most pronounced in the intestinal stem cell compartment, greatly facilitates oncogenic transformation following *Apc*-inactivation by alleviating *Apc*-loss induced pro-apoptotic signals [28]. Of note, ectopic expression of *Bcl-2* in more differentiated cells also rendered these cells susceptible to generating oncogenic outgrowth following Wnt pathway activation. Of further interest is the high level of telomerase activity detected upon β-catenin activation, potentially explaining why Wnt active cells are more permissive to tumorigenesis when mutations occur [29]. Besides the gastrointestinal tract, other tumor types have shown cellular and even regional specificity for Wnt mediated transformation. For instance, in mouse models of medulloblastoma it was reported that activating mutations in β-catenin only confer a proliferative advantage to dorsal brainstem progenitor cells but not in cerebral granule neuronal precursors (CGNPs) [30].

### 3.2. Wnt Active Cancer Stem Cells

There are several lines of evidence to support the notion that high Wnt activity identifies cancer cells endowed with stem cell capabilities. In CRC, despite the presence of mutations that constitutively turn on Wnt signaling, remnant regulation of the transduction cascade occurs which results in various degrees of Wnt signaling output within a clonal population of cancer cells. Using a Wnt reporter construct, we have reported that human colorectal CSCs can be defined on the basis of high Wnt signaling activity, and these cells preferentially localize to a myofibroblast niche [31]. Already in CRC precursor lesions, high Wnt activity defines the CSC fraction [32]. More recently, the use of a Wnt reporter has linked Wnt activity and the CSC phenotype across multiple human tumor types, including lung, gastric, and breast cancers [33]. In addition, various cell surface markers that have been used to enrich for CSC populations relate to Wnt activity or are canonical Wnt target genes (Table 1). As an example, elevated levels of Lgr5 expression can be detected in CRCs and tumor cells with the highest level of Lgr5 have been shown to behave as functional CSCs both in humans and mice [34,35]. Similarly, Wnt target gene CD44 expression also identifies the CSC fraction both in primary and metastatic CRC, breast cancer, and many other malignancies [36,37,38].

As we have summarized, Wnt activating mutations have been shown to be essential for the initiation of several tumor types and tumor maintenance is also dependent on Wnt activity irrespective of the presence of additional driver mutations [11]. Interestingly, in the case of CRC patients, where constitutively activating mutations of the Wnt pathway are clonally present within all tumor cells, such tumors often still reveal a significant degree of regulation of the pathway [55]. Furthermore, truncating events targeting the *APC* gene rarely result in a complete null genotype. Instead, there seems to be a particular selection for alleles that retain some level of β-catenin regulation. This has formed the basis for the “just-right” hypothesis, which postulates that a finely tuned β-catenin/Wnt activity level is required to optimally endow incipient tumor cells with tumorigenic potential. As we will elaborate below, there are various levels at which regulation of Wnt activity operate. Both intrinsic as well as microenvironmental regulation have been reported but altogether they seem to converge towards a similar phenotypic quality: tumor cells displaying high Wnt activity are functionally related to cancer stem cells (CSCs).

## 4. Regulation of Wnt Activity and CSC Properties

**Figure 2:**
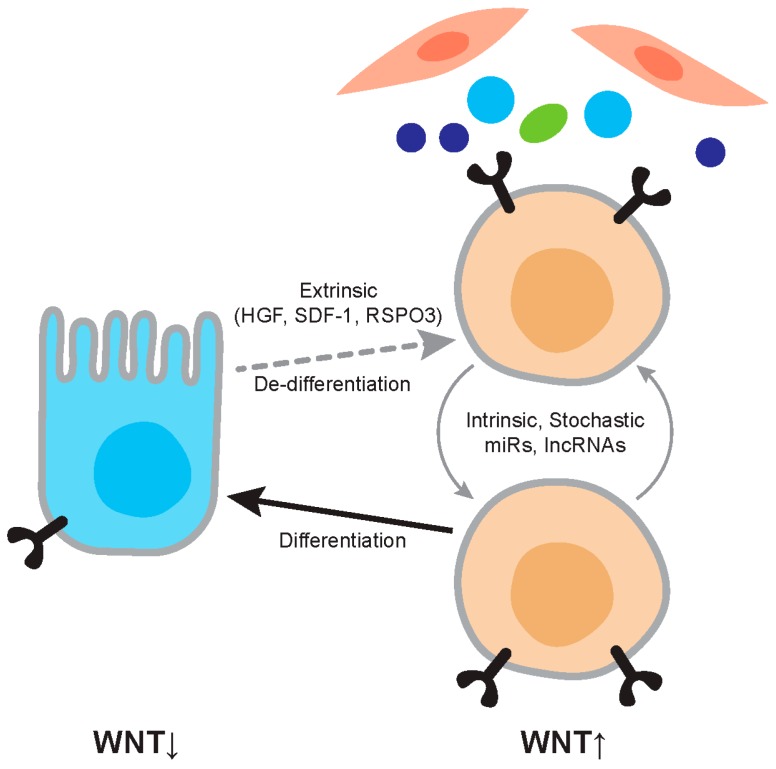
Regulation of Wnt activity in cancer. The figure depicts the relationship between differentiated tumor cells and cancer stem cells. Differentiated cells are relatively Wnt-low and cancer stem cells relatively Wnt-high. Wnt activity levels are dependent on extrinsic factors such as Hepatocyte Growth factor (HGF) and RSPO3, and on intrinsic factors such as mutation and expression levels of microRNAs (miRs). The cancer stem cell phenotype is not stable and differentiation and dedifferentiation are ongoing processes.

**Figure 3:**
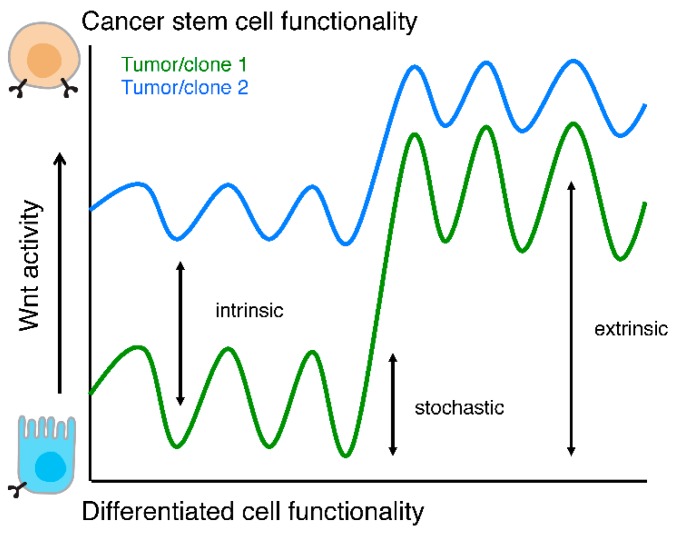
Wnt activity levels and functionality. Schematic representation of cellular functionality as a function of Wnt activity levels. Two tumors or clones are depicted (green and blue lines) to highlight that intrinsic Wnt activity levels, stochastic fluctuations, and responses to extra cellular stimuli are different between two (epi)genetically distinct populations.

The key hallmark of the CSC concept is that genetically identical cells, belonging to the same malignant clonal population, display distinct tumor propagating features. As we discussed, this distinction between the cancer growth-driving, stem cell-like population, and the more differentiated and indolent cells is intimately related to Wnt signaling levels, with the former cells showing high activity of this pathway. An important focus of research is to establish what signals maintain and install these differential Wnt activity levels and thereby regulate the CSC population (Figure 2). This is an especially intriguing topic of research as many malignancies, and also the cancers in which tumorigenic cells can be identified based on Wnt signaling activity-display genetic aberrations, directly enhance activity of this pathway. As we discussed above, over 80% of CRCs display inactivating *APC* mutations that inhibit β-catenin degradation and thereby enhance Wnt pathway activity. Yet, despite this global increased level of activity in all cancer cells, additional regulation occurs, and cells with distinct activity levels can be identified and associated with clear biological significance. This regulation takes place at several levels and displays clear resemblance to regulation of the Wnt pathway in normal stem cell compartments (Figure 2 and Figure 3).

### 4.1. Microenvironmental Regulation

#### 4.1.1. Wnt Ligands

In normal tissues, secreted Wnt ligands bind Frizzled receptors in conjunction with Lrp co-receptors to activate intracellular Wnt signaling in a phosphoprotein Disheveld (Dsh) mediated fashion. Following Wnt ligand binding, the β-catenin degradation complex, comprising AXIN, APC, and GSK3β, translocates to the plasma membrane where its activity is inhibited by several mechanisms: the stability of components of the complex is diminished and GSK3β kinase activity is reduced resulting in relative stabilization of β-catenin. β-catenin accumulates, translocates to the nucleus, and enhances Wnt target gene expression in a TCF/Lymphoid Enhancer-Binding Factor (LEF) dependent manner.

Sources of Wnt ligands in normal tissues are the stem cell-supporting cells comprising the cellular niche. For example, Paneth cells in the intestine produce Wnts in support of the crypt base columnar stem cell population. Additionally, in the hematopoietic stem cell niche Wnt ligands are pivotal in the regulation of stem cell function and hematopoiesis [56]. The importance of Wnt ligands in the maintenance of CSCs has been established in a large variety of malignancies.

In estrogen receptor negative (ER negative) breast cancers Wnt3a has been demonstrated to increase the sphere forming ability [57]. Similarly, both Wnt1 and Wnt3a support stem-like cells in glioblastoma multiforme (GBM) [58]. The relevance of Wnt ligands in stem cell function was further supported by the notion that periostin (POSTN), an extracellular matrix component, facilitates metastasis formation by locally sequestering Wnt ligands that support Wnt activity in metastatic breast CSCs. Conversely, non-canonical Wnt signaling induced by Wnt5a constrains canonical Wnt activity and inhibits stem cell properties in breast cancer. Heterozygous loss of the WNT5A gene was associated with poor disease outcome in this malignancy. This highlights the delicate balance of pro- and anti-stem cell properties of the Wnt ligand family. Furthermore, work from the Weinberg laboratory revealed that both the canonical and non-canonical Wnt pathways cooperate with TGFβ signaling in not only the maintenance, but intriguingly also the induction of stem cell properties in breast cancer cells [59]. These pathways can be activated in an autocrine fashion as this work demonstrates, although it is expected that other sources in the tumor environment contribute significantly as well. Inhibition of these pathways results in decreased tumorigenicity and metastatic properties [59], providing a rationale for therapeutic targeting of these signals.

#### 4.1.2. R-spondin-LGR5-Rnf43 Signaling

Lgr5 is both a Wnt signaling component as well as a prominent Wnt target gene. Lgr5 is a cell surface G-protein coupled receptor (GCPR) that binds R-spondins, which activate the Frizzled-LRP5/6 complex together with Wnt ligands. As a result, both Lgr5 and R-spondins augment Wnt ligand mediated Wnt signaling. Further evidence has indicated that R-spondin proteins inhibit the E3 ubiquitin ligases Rnf43 and Znrf3 that cause stabilization of Frizzled receptors, thereby enhancing Wnt signaling (Figure 1B).

These insights suggest that the R-spondin-Lgr5-Rnf43 axis enhance Wnt activity and thereby likely promote stem cell properties. Indeed, Lgr5 has been reported to be a CSC marker in many malignancies which might both reflect the high Wnt activity levels of these cells and also the functionality of Lgr5 in promoting stem cell functions. In agreement, in breast cancer the overexpression of Lgr5 or its inhibition results in increased and decreased stem cell functionality, respectively [60]. Also, in neuroblastoma [61] and Ewing sarcoma [62] Lgr5 potentiates Wnt activity and increases malignant potential. In contrast, in CRCs it was described that the expression of R-spondin 2 (RSPO2) is frequently suppressed by methylation of the locus [63]. Surprisingly, it was demonstrated that RSPO2 suppresses Wnt activity in an Lgr5 dependent fashion in the vast majority of CRC lines, and that Lgr5 expression reduces stem cell properties [63]. These insights establish that both R-spondins and Lgr5 can also display tumor suppressive features dependent on the context. The ambiguous associations found between Lgr5 expression and outcome in CRC might be explained by these findings [64,65,66,67,68,69].

#### 4.1.3. Cytokine Signaling (HGF, Osteopontin, SDF-1)

The tumor microenvironment is characterized by an influx of activated cell types, including cancer-associated fibroblasts (CAFs) and various types of immune cells. These cells, together with the cancer cells, produce a plethora of cytokines and signaling molecules of which many have a direct impact on Wnt signaling activity. As we described above, in CRC it was long known that despite activating mutations in the Wnt pathway that occur early in tumor development, such as *APC* truncating mutations or β-catenin/*CTNNB1* stabilizing mutations, heterogeneous Wnt activity levels can be detected as evidenced by various degrees of nuclear localized β-catenin in individual cancers. This suggests that Wnt signaling can be modulated by extrinsic features in otherwise homogenous populations of cancer cells all harboring Wnt activating mutations. This notion became known as the “β-catenin paradox” and in recent years many factors have been identified that are accountable for this observation [55]. In the original description of the β-catenin paradox it was noted that the cells displaying evident nuclear localized β-catenin were distributed non-randomly and were especially abundant in the invasive front and in areas in close proximity to myofibroblasts [55]. Indeed, as we now know factors secreted by myofibroblasts, including Hepatocyte Growth factor (HGF), Osteopontin (OPN), and stromal-derived factor 1α (SDF1α), enhance the activity of the Wnt pathway in CRC cells [31,52]. This is mediated, for example, by inhibition of GSK3β following serine 9 phosphorylation and by direct stabilizing of β-catenin. The cytokine mediated increase in Wnt activity is of great functional relevance as it is required for cancer stem cell functions such as proliferation and tumorigenicity. More strikingly, activation of the Wnt pathway by the aforementioned cytokines can install a CSC phenotype in more differentiated tumor cells. This insight revealed that the CSC hierarchy is less stringent than anticipated before and dictated by modulation of Wnt signaling levels by the tumor microenvironment. Similarly, IL17A, another cytokine produced by CAFs, increases the tumorigenic CRC cell population expressing the Wnt target and CSC marker CD44 [70]. Intriguingly, IL17A is predominantly expressed in CAFs that have been exposed to chemotherapy, and therefore this cytokine might be extremely important in driving disease relapse.

### 4.2. Intrinsic Regulation

Besides regulation of the Wnt pathway that is exerted by extracellular factors additional cancer cell intrinsic features can also contribute to Wnt modulation and thereby CSC biology. These factors are genes or microRNAs (miRs) that are up- or down-regulated following genetic aberrations that occur during tumor development and might be related to long-lasting input from the microenvironment, or potentially represent epigenetic features associated with the cell of origin of the malignancy. In many cases the exact mechanism by which these internal regulators of Wnt activity are established remains unknown, but their downstream effect of Wnt signaling is evident. Knowledge of these factors is critical as they are both informative in elucidating the intricacies of the Wnt cascade in cancer but also might constitute important therapeutic targets. In contrast to the extracellular Wnt regulators, the means by which these types of regulators dictate the CSC vs non-CSC distinction is less clear-cut. However, it can be envisioned that these features increase or decrease global Wnt activity levels thereby impacting on the proportion of CSCs in that cancer (Figure 3). At the single cell resolution the CSC properties in this model are either defined by external factors, such as those listed above, or by stochastic fluctuation in intrinsic Wnt regulatory molecules. Indeed, CSC identity is partially defined by stochastic processes [71] and CSC content of malignancies is dependent on the genetic characteristics of the malignancy [72] (Figure 3).

With respect to intrinsic regulators of Wnt activity levels, it was recently established that proliferative cell nuclear antigen (PCNA) associated factor (PAF) is upregulated in breast cancer as compared to healthy mammary tissue [73]. PAF is required for breast CSCs and it exerts its function via activation of the Wnt/β-catenin pathway probably via a direct interaction with the β-catenin transcriptional complex as has been demonstrated before [73,74]. In CRC, it has been reported by us, and others, that key target genes of the pathway are methylated during disease progression, including *AXIN2*, *APCDD1,* and *DKK1* [68,75,76]. Intriguingly, all these genes are negative regulators of the pathway and it is likely that silencing contributes to enhanced Wnt activity and potentially the expansion of the CSC pool [68]. Similar observations are reported in many different malignancies [77,78,79,80]. These data support a model in which epigenetic alterations modify the basal Wnt activity levels within malignant cell populations, and thereby very likely also CSC properties, although this requires further experimentation.

Similarly, a large number of miRs have been implicated in CSC maintenance via modulation of the Wnt signaling axis. To name a few: miR-21 has been established to be upregulated in CRC and promotes stem cell properties. Mechanistically, miR-21 suppresses transforming growth factor β receptor (TGBR2), which results in activation of the Wnt pathway, and increases c-Myc and cyclin D1 levels [81,82]. miR-451 expression is associated with a decreased tumor initiating ability of CRC cells. Suppressed miR-451 levels result in increased macrophage migration inhibitory factor (MIF) that activates Cyclooxygenase-2 (COX-2), which has been associated with Wnt pathway activation [83,84]. More direct interactions of miRs with Wnt pathway components are reported as well. In prostate cancer, β-catenin was shown to be a direct target of miR-320, and overexpression of this miR results in reduced Wnt activity and a reduction in CSC marker expression [85]. miR-214 enhances CSC properties in lung cancer by directly modulating the levels of the negative Wnt regulator β-catenin interacting protein 1 (CTNNBIP1). Furthermore, in breast cancer a whole series of miRs have been implicated in the regulation of Wnt signaling and CSC properties, possibly reflecting the lack of genetic activation of the pathway in this disease. For example, miR-142 is highly expressed in breast CSCs, but interestingly absent from normal mammary stem cells [86,87]. It activates Wnt signaling by directly targeting APC. Moreover, miR-142 is a direct Wnt target and, as a result, this miR is critical in maintaining and self-sustaining the Wnt-activating loop with critical relevance for breast CSCs [86].

Another example of non-coding RNAs in Wnt signaling regulation relevant for CSC biology comes from hepatocellular carcinoma (HCC). In HCC long noncoding RNA (lncRNA) lncTCF7 that is highly expressed in this malignancy is required for CSC maintenance [88]. lncTCF7 activates Wnt signaling by recruiting the Switch/sucrose nonfermenting (SWI/SNF) complex to the TCF7 promoter [88].

To conclude this section, we wish to highlight that direct genetic events also impact on the structure of the CSC hierarchy by means of Wnt pathway modulation. Obviously, it is expected that specific Wnt activating mutation results in distinct degrees of Wnt pathway activation, and that these different basal activity levels impact on stem cell properties in a similar fashion, as has been detected along the intestinal tract [19]. Unfortunately, to our knowledge no detailed analysis of this concept has been presented to date. In addition, other mutations that indirectly impact on Wnt pathway activity, and thereby CSC properties, are also expected to contribute. In this respect, it has been reported that a rarely detected oncogenic Epidermal growth factor receptor (EGFR) variant (EGFRvIII) promotes CSC function through Wnt pathway activation, although the molecular mechanism remains uncharacterized [89].

## 5. Therapeutic Targets

As we have discussed in this review, there is an extensive body of evidence supporting the concept that Wnt signaling defines CSCs and is key to their biology. Hence, targeting this pathway is an attractive therapeutic target for cancer. However, the characteristics of the signal transduction cascade, and the molecular mechanisms by which its activity is regulated, make it a very difficult task. The field has proven to be very good in generating inhibitors of kinase activity and antibodies that bind either growth factors or their receptors. Drugs that fall in these categories have been proven to be clinically most successful. Unfortunately, the regulation of the Wnt pathway relies heavily on protein-protein interactions, and blocking these has proven to be much more challenging. Furthermore, kinases involved in the pathway predominantly exert an inhibitory effect (e.g., GSK3β is inhibited by phosphorylation, and phosphorylation of β-catenin by GSK3β primes it for degradation), therefore devising direct kinase inhibitors of pathway components is problematic.

Yet, accumulating knowledge in the regulation of the pathway has led to novel putative targets that can, in principle, be effectively modulated by drugs. For example, tankyrase inhibitors prevent poly(ADP-ribosyl)ation-dependent AXIN degradation, thereby promoting β-catenin destabilization. Critically, these agents also exert their Wnt inhibitory properties in the presence of *APC* mutations. Indeed, tankyrase inhibition has been shown to reduce the growth of *APC* mutant CRC tissue in xenograft models [90], as well as tumor development in an *Apc*-loss driven mouse model [91]. This reduced growth is accompanied by induction of differentiation and reduced clonogenicity, thereby corroborating the importance of the Wnt pathway in CSC regulation in this malignancy [90]. Furthermore, combinations of tankyrase inhibitors with other targeted drugs or chemotherapy are reportedly effective in preclinical CRC models [92,93]. Tankyrase inhibition has also been shown to reduce the growth of breast cancer cells that are *APC* wild type only under serum deprived conditions [94], induce apoptosis and differentiation in osteosarcoma cell lines [95], and inhibit stem cell properties and migration of CSC-like cells in neuroblastoma [96]. 

Another Wnt component in the spotlight for Wnt inhibitor development is Dvl. The PDZ domain of Dvl acts as a critical mediator of the Dvl-Frizzled interaction and downstream transduction of the Wnt signal. PDZ domain inhibitors have been discovered that inhibit the Wnt pathway [97,98]. Alternative compounds that target Wnt signaling are porcupine inhibitors that prevent the secretion of Wnt ligands by inhibiting the palmitolation of these molecules, which is a required process for effective shedding [99,100]. The LGK947 compound is currently in clinical phase 1 testing for Wnt driven cancers (clinicaltrials.gov). The mechanism suggests that porcupine inhibition will be mainly effective in cancers that are Wnt dependent but do not harbor downstream Wnt activating mutations in genes such as *APC* or *CTNNB1*. Instead, cancers that rely on upstream Wnt activating events, including the recently reported fusion events involving RSPO2 and RSPO3 in a small percentage of *APC* wild type CRCs [5,101] or RNF43 mutant pancreatic cancers [102]. Indeed, the porcupine inhibitor ETC-159 has demonstrated clear efficacy in these cancers [103]. In addition, the porcupine inhibitor C59 has demonstrated activity against Rnf43 and Znrf3 double mutant intestinal neoplasia, thus suggesting that paracrine Wnt signaling is indeed essential for tumor growth driven by aberration in these Wnt cascade components [103]. Alternatively, CRCs presenting with PTPRK-RSPO3 might be effectively treated with R-spondin blocking antibodies [6]. Intriguingly, Wnt inhibition in these cancers induces persisting differentiation and downregulation of CSC markers *ASCL2* and *LGR5*. Similarly, OncoMed Pharmaceuticals reported anti-RSPO treatment of RSPO producing ovarian, colon, lung, and pancreatic cancers attenuated cancer growth and was again associated with reduced expression of CSC marker genes [42]. This same company generated two distinct agents that interfere with Wnt ligand induced Frizzled signaling. Firstly, the anti-Fzd7 monoclonal antibody targets a range of Frizzled receptors and has shown anti-tumor efficacy in a series of preclinical tumor models including pancreas, breast, and lung cancer [104]. Secondly, Fzd8-Fc consists of a human Frizzled 8 receptor coupled to a human IgG1 Fc fragment [105]. This agent sequesters native Frizzled 8 ligands resulting in the inhibition of ligand mediated Wnt signaling, and has been suggested to reduce CSC numbers and tumor growth in several cancer types [105]. Phase 1 clinical trials with anti-Fzd7 and Fzd8-Fc are currently ongoing.

As indicated, upstream targeting of the Wnt pathway is unlikely to show efficacy in cancers with genetic activation of the pathway by mutations in more downstream components. However, a multitude of strategies have been employed to impede β-catenin activity. PRI-724 disrupts the complex β-catenin forms with CBP (cyclic AMP response element binding protein), which reduces the expression of a subset of Wnt target genes that are particularly important for (cancer) stem cell proliferation [106,107]. Several phase I/II trials are currently ongoing in hematological malignancies, pancreatic cancer, and colon cancer [108]. Recently, another promising compound (LF3) that directly inhibits the β-catenin/TCF4 interaction has been reported [109]. LF3 treatment of colon, head, and neck cancer cells results in the suppression of Wnt activity and reduced self-renewal properties of CSCs. Finally, this compound induced differentiation of colon cancer xenografts, which was also accompanied by growth suppression [109]. Further (pre-)clinical testing of this drug is warranted and is anxiously awaited.

Additional opportunities to reduce Wnt pathway activity in cancer focus on targeting the growth factors and cytokines that modulate the pathway. Examples of these include HGF inhibition, which reportedly reduces colon cancer tumor growth [110], potentially by reducing Wnt activity, and thereby CSC frequencies [31,111]; or similarly, direct targeting of c-Met, the HGF receptor, that reduces CSC numbers in head and neck squamous cell carcinoma (HNSCC) [112]. It was reported that in this malignancy c-Met activates the Wnt pathway by inducing upregulation of FZD8. Disruption of c-Met activity by pharmacological inhibition reduced Wnt levels and consequently eliminated HNSCC CSCs. Further evidence to support the notion that Wnt signaling is critical for stem cell functions in HNSCC comes from the study of the secreted Frizzled related protein 4 (sFRP4) [113]. Treatment of two HNSCC cell lines with the Wnt antagonist sFRP4 resulted in reduced Wnt activity, impaired clonogenicity, decreased expression of stem cell associated markers, and critically increased sensitivity to cisplatin [113]. Hence, it is worth further exploring the therapeutic potential of naturally occurring Wnt antagonists. Critically, it was recently reported that autocrine inactivation of Wnt signaling by DKK1 can induce, in some cases, a quiescent and immune evasive state [114]. These latency competent cancer (LCC) cells have the ability to be reactivated after an extensive period of time. Therefore the long-term benefits of Wnt signaling impairments remains to be established for all of the above indicated therapeutic strategies.

## 6. Synopsis

As our review of the literature advocates, there is an overwhelming amount of evidence to support the critical role of the Wnt pathway in CSC biology, thereby mirroring the even larger body of studies advocating the critical function of this signaling cascade in normal stem cells. Wnt pathway activity is associated with the stem cell population in many malignancies and the most prominent CSC markers used to identify this population are, in fact, direct Wnt target genes including *LGR5*, *CD44,* and *PROM1* (CD133) [51,115,116]. Furthermore, in the vast majority of model systems inhibition of Wnt signaling, either directly or by interfering with pathways that promote Wnt activity, results in decreased stem cell numbers and impaired tumorigenicity. Interestingly, this is both the case in malignancies that are characterized by Wnt activating mutations as well as in malignancies that rely on extracellular input for Wnt cascade activity. Conversely, increased Wnt activity is almost invariably associated with increased stem cell potential and more aggressive cancers. Hence, the development of agents that impair Wnt signaling are at the center stage in drug development, although it remains a significant challenge given the peculiarities of the transduction route as we have argued. However, the central role of Wnt in normal stem cell biology will potentially limit the tolerance to these agents. Fortunately, due to robust wiring of normal stem cells and high redundancy of signaling input in stem cell niches this appears to be less of a problem than initially anticipated. For several Wnt inhibitors significant therapeutic windows have been reported [117,118]. It is expected that these agents will be further improved in the coming years and that therapeutic efficacy will be demonstrated in selected patient populations to help limit the burden of disease.

## Figures and Tables

**Table 1:** Wnt activity defines cancer stem cells (CSCs) in multiple malignancies. Below is an overview of the relationship between Wnt signaling activity and CSC properties in various malignancies. Of note, this is by far not a complete overview but only intended to provide several key examples.

Marker	Tumor type	Reference
Wnt activity		
*TCF Reporter*	Colon, Ovarian, Gastric, Lung	[31,33,39,40]
*β-catenin*	Skin	[41]
*RSPO*	Colon, Ovarian	[6,42]
*BCL9*	Colon	[43]
Wnt target gene		
*Lgr5*	Colon, Gastric	[34,35,44]
*CD24*	Pancreas, Colon	[45]
*Prom1*	Colon, Brain, Pancreas	[46,47,48,49]
*CD44*	Colon, Breast, Liver, Pancreas, Prostate	[36,37,50,51,52]
*ALDH1*	Colon, Breast	[53,54]
